# Preparation of an Ultrafiltration (UF) Membrane with Narrow and Uniform Pore Size Distribution via Etching of SiO_2_ Nano-Particles in a Membrane Matrix

**DOI:** 10.3390/membranes10070150

**Published:** 2020-07-10

**Authors:** Bushra Khan, Sajjad Haider, Rooha Khurram, Zhan Wang, Xi Wang

**Affiliations:** 1 College of Environmental and Energy Engineering, Beijing University of Technology, Beijing 100124, China; bushrakhan01@hotmail.com (B.K.); sajjadsaleem266@hotmail.com (S.H.); rooha_khurram@hotmail.com (R.K.); 2 School of Humanities and Social Sciences Macao, Polytechnic Institute, Macao 999078, China

**Keywords:** polyvinylidene fluoride, hydrofluoric acid, antifouling, hydrophilicity, silicon dioxide nano-particle

## Abstract

The UF membrane with a narrow and uniform pore size distribution and a low tendency to foul has significant applications in wastewater treatment. A major hindrance in the preparation of the UF membrane with these features is the lack of a scalable and economical membrane fabrication method. Herein, we devise a new strategy to prepare a high-quality polyvinylidene fluoride/polymethyl acrylate/cellulose acetate (PVDF/PMMA/CA) blend UF membrane via a combination of the etching mechanism with the traditional Loeb–Sourirajan (L-S) phase inversion method. Different concentrations of silicon dioxide (SiO_2_) nanoparticles (NP) in the membrane matrix were etched by using a 0.2 M hydrofluoric acid (HF) solution in a coagulation bath. This strategy provided the membrane with unique features along with a narrow and uniform pore size distribution (0.030 ± 0.005 μm). The etched membrane exhibits an increase of 2.3 times in pure water flux (PWF) and of 6.5 times in permeate flux(PF), with a slight decrease in rejection ratio (93.2% vs. 97%) when compared to than that of the un-etched membrane. Moreover, this membrane displayed outstanding antifouling ability, i.e., a flux recovery ratio (FRR) of 97% for 1000 mg/L bovine serum albumin (BSA) solution, a low irreversible fouling ratio of 0.5%, and highly enhanced hydrophilicity due to the formation of pores/voids throughout the membrane structure. The aforementioned features of the etched membrane indicate that the proposed method of etching SiO_2_ NP in membrane matrix has a great potential to improve the structure and separation efficiency of a PVDF/PMMA/CA blend membrane.

## 1. Introduction

UF membranes have been widely used for water purification and they can successfully remove macro-molecules (such as proteins) from contaminated water [1].

This is mostly due to their unique properties, such as high separation efficiency, low driving pressure, and low operating temperature. It is a renowned fact that high-efficiency UF operations must possess elevated flux, high retention rate, and excellent antifouling properties, which is mostly effected by pore structure and the surface properties of the UF membranes [2,3]. The membranes which possess a large number of pores with narrow pore size distribution can exhibit superior filtration efficiency [4,5]. Compared to traditional separation methods, membrane-based processes can substantially save energy and reduce capital costs [6]. 

Recently, many approaches have been put forth by researchers to improve the pore size and porosity of membranes by introducing a chemical reaction in the membrane fabrication process. For instance, Sen Yang et al. [7] controlled the pore size and porosity of PAN membranes using calcium chloride (CaCl_2_) as an additive in casting solution and sodium carbonate (Na_2_CO_3_) in a coagulation bath. They observed that the prepared membranes exhibited higher permeability and a bigger pore size due to the chemical reaction that occurred between CaCl_2_ and Na_2_CO_3_ in the coagulation bath. Meanwhile, Sen Yang et al. [8] also used a gas–liquid interfacial chemical reaction to control the pore size and porosity of a membrane. Wang et al. [9] used a chemical reaction between acetic acid (CH_3_COOH), an additive of casting solution, and 2 wt% of Na_2_CO_3_ in the coagulation bath. They found that as a result of the chemical reaction, CO_2_ was produced, which played an important role in the improvement of membrane porosity and performance. In our laboratory’s previous work, the pore size distribution of the PVDF/PMMA/CA blend membrane was improved by a reaction between different organic acids in the casting solution and sodium carbonate/bicarbonates in the coagulation bath [10].

The polyvinylidene fluoride (PVDF) polymer is one of the most promising candidates for the UF membrane preparation due to its high durability, excellent chemical stability and biological resistance. However, PVDF is susceptible to surface fouling due to its hydrophobic nature, which declines the flux [11,12] and limits the use of PVDF membranes in membrane-based filtration processes. Thus, the hydrophilic group must be introduced into the PVDF membrane material in order to improve its antifouling characteristics [13]. To address this issue, several methods have been investigated to ameliorate surface hydrophilicity and antifouling property of PVDF membranes, such as a coating of antifouling substances on the polymer surface [14,15,16], blending with hydrophilic group-containing materials, incorporation of hydrophilic additives and inorganic nanoparticles in polymer matrixes during the membrane fabrication process [17,18,19,20,21], etc.

The results of blend methods for PVDF/ PV, PVDF/PS, PVDF/PAN, PVDF/PMMA/CA, and PVDF/SPS systems showed that the blend method is a convenient, widespread and effective method without destroying the structure of the polymer chains [22,23,24] in order to prepare a high-quality antifouling PVDF membrane [25,26,27,28,29,30]. For example, when high-quality hydrophilicity materials, CA and PMMA, are blended with PVDF, the hydrophilicity and antifouling characteristics of a PVDF membranes can be improved, their lifetime can be prolonged and membrane operating costs can be reduced [31].

In the traditional L-S phase-inversion process, many chemical reagents such as lithium chloride (LiCl) [32], lithium perchlorate (LiClO_4_) [33], ethanol and mixtures of LiCl/water and LiCl/ethanol [34] were introduced in casting solutions, while some inorganic salts (Na_2_CO_3_/K_2_CO_3_ [10], NaHCO_3_/ KHCO_3_ [35] were introduced in the coagulation bath to react with certain components (different organic acids) in the casting solution to prepare the blend PVDF/PMMA/CA membrane [36]. Despite their successes, the preparation of the blend PVDF/PMMA/CA membrane with a narrow and uniform pore size distribution and low tendency to foul is still a challenging task. To overcome this problem there is a need to explore innovative strategies which can decrease the energy cost, increase the clean water productivity, and enhance the antifouling property of membranes.

**Figure 1:**
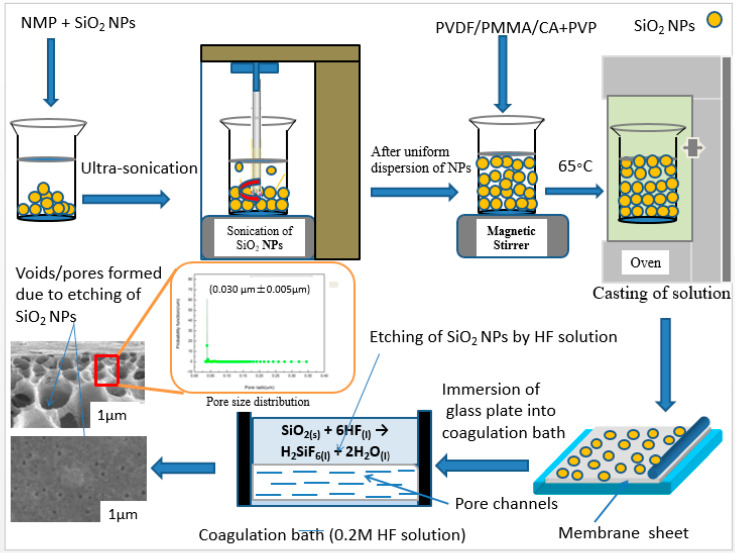
The schematic illustration of the overall preparation process of the etched UF membranes.

In the current study, we report a facile and simple strategy for the preparation of the blend PVDF/PMMA/CA membrane to improve the aforementioned problem. The proposed synthetic method refers to control the membrane pore size distribution by the selective etching of SiO_2_ nanoparticles in the membrane matrix by using hydrofluoric acid (HF) in a coagulation bath combined with the traditional L-S phase inversion method. As shown in Figure 1, SiO_2_NPs were etched in membrane matrix by 0.2 M HF solution to generate the pores throughout the membrane structure. During the membrane formation process, the concentration of hydrofluoric acid (HF) was kept constant. This strategy provided the resulting PVDF/PMMA/CA blend UF membranes with a narrow and more uniform pore size distribution and highly enhanced permeability.

To the best of the authors’ knowledge, the etching of SiO_2_ NPs in s membrane matrix combined with the L-S phase inversion method to prepare PVDF/PMMA/CA blend membranes has not been reported yet in the literature. This technique may be a feasible approach for the preparation of highly porous PVDF/PMMA/CA membranes to achieve a uniform pore size distribution, superior antifouling property and high permeability, which are essential for membranes to satisfy the requirement of industrial separation processes.

## 2. Experimental Section

### 2.1. Materials

The membrane materials PVDF/PMMA(Mw = 4000–5000)/CA-398 were obtained from Shanghai 3F New Material Ltd., China, η = 1.4–1.9/Beijing Organic Chemical Engineering Factory/Britain Nelsons Acetate. Solvents: N-methyl-2-pyrrolidone (NMP)/HF were procured from Tianjin Fuchen Chemical Reagent Factory, China. Additive PVP was purchased from Beijing Chemical Engineering Factory. Bovine serum albumin (BSA) (Mw = 67,000 Da), used as a reagent for determining the observed retention of the membrane, was acquired from Beijing Microorganism Culture Medium Manufacturing Corporation China and its isoelectric point of pH is 4.8. Deionized-water was used in all experiments.

### 2.2. Preparation of PVDF/PMMA/CA Blend Membranes 

The detailed fabrication procedure is shown in Figure 1. Initially, different concentrations of SiO_2_ NPs (0.3, 0.5, 0.7 and 1 wt%) were dispersed into a specific amount of NMP via vigorous sonication for 1 h. After attaining the uniform dispersion of NP, the stoichiometric amounts of PVDF, PMMA and CA were dissolved in the solutions according to a certain ratio (11.2:2.4:2.4) under intensive stirring. After complete dissolution of the polymers, a measured amount of PVP (3 wt%) was added and the resulting solution was intensively stirred for 2 h in order to achieve a homogeneous mixture of all ingredients. Then the casting solutions were placed in the vacuum oven at 65 °C for 4 days. 

Later, the casting solutions were cast on clean glass plates with a casting blade under 25 °C and 30% humidity, and the plates were gently immersed into a coagulation bath containing DI-water/0.2 M HF solution at 25 °C for 30 mins. Finally, the membrane were stored in DI-water at room temperature for 2 days prior to filtration test in the stirred cross flow cell. The resultant membranes with DI-water as coagulant were M@SiO_2(0)_, M@SiO_2(0.3)_, M@SiO_2(0.5)_, M@SiO_2(0.7)_ M@SiO_2(1)_ while the membranes with HF solution as coagulant were named M@SiO_2(0)_HF, M@SiO_2(0.3)_HF, M@SiO_2(0.5)_HF, M@SiO_2(0.7)_HF and M@SiO_2(1)_HF, respectively.

#### 2.2.1. Design of Coagulation Bath for Etched Membranes

The coagulation bath was comprised of 0.2 M of HF solution for the etched membranes.

#### 2.2.2. Basic Chemical Reaction

On immersing cast films into the coagulation bath, the following chemical reaction occurred between SiO_2_ NPs in the membrane matrix and 0.2 M HF solution in the coagulation bath.

SiO_2(s)_ + 6HF_(l)_ → H_2_SiF_6(l)_ + 2H_2_O_(l)_

### 2.3. Characterization of Etched UF Membranes 

SEM images of all the synthesized membranes were obtained by using a scanning electron microscopy (SEM, S-4800, Hitachi Limited Inc., Tokyo, Japan). Snapping of the membranes was completed under liquid nitrogen to give a clean and concordant cut. Double-sided adhesive tape was used to mount the membrane samples on sides of brass plate and then samples were sputtered with Au before observation.

The sessile-drop method (Zwick/Roell BL-GRS500N) was utilized to measure the water contact angles (WCA) at 25 °C and 50% relative humidity. Before measurements, all the membrane samples were dried for 24 h at room temperature. In order to minimize the systematic error and to attain the accuracy, five measurements on different locations of each sample were taken and the mean of resulting values was computed. 

The liquid–liquid displacement technique was applied to analyze the pore size distribution by utilizing n-butyl alcohol–water as a solvent pair [37]. The pore size distribution functions were calculated by using Equations (1) and (2):

(1) $$r=\frac{2\sigma \cos\theta }{P}$$

(2) $$f(r)=\frac{Pi(P_{i-1}J_{i}-P_{i}Ji_{-1})}{\left(r_{i-1}-r_{i})P_{i-1}\Sigma^{m}{}_{{}_{i-1}}\frac{P_{i}}{P_{i-1}}(P_{i-1}J_{i}-P_{i}J_{i-1}\right)}$$

where, r, σ, *θ* and *J_i_* represent the pore radius, the surface tension of n-butyl alcohol–water, the polymer-n-butyl alcohol contact angle, and the flux measured at the *i^th^* increment where the applied pressure is *P_i_,* respectively [38,39]. The dry–wet method was used to measure the porosity of the membrane samples by using Equation (3):

(3) $$P_{0}=\frac{W_{2}-W_{1}}{V_{0}.d_{water}}$$

where *P_o_* is the porosity of the membrane (%), *W_2_* is the weight of the wet membrane (g), *W_1_* is the weight of the dry membrane (g), *V_o_* is the volume of the membrane (cm^3^) and *d_water_* is the water density at room temperature (g·cm^−3^).

### 2.4. The Performance of Etched UF Membranes

Permeation tests were performed using a cross-flow stirred cell having an effective area of 24 cm ^2^. One g/L BSA protein was used as a feed solution at 0.1 M Pa and 25 °C. Rejection was checked on UV–Vis spectrophotometer (Shimadzu, Japan) at a wavelength of 280 nm. The equations employed to compute the resultant flux and rejection are as follows [2,10]:

(4) $$J_{P}=\frac{\Delta V}{A\Delta tP}$$

where *J_P_*, ∆V, ∆*t* (h), *P* and A (m^2^) denote the permeate flux (L/(m^2^ h)), the permeate volume (L), the filtration time interval (h), the driving pressure (Mpa) and the effective area (m^2^), respectively.

(5) $$R=(1-\frac{C_{f}}{C_{p}})\times 100\%$$

where the feed solution and permeate concentrations are denoted by C_f_ (g/L) and C_p_ (g/L), respectively.

### 2.5. Antifouling Performance Measurements

In order to study the antifouling performance of membranes, BSA as a typical foulant was utilized as feed solution. Firstly, to measure the pure water flux (*J_W_*) of the membrane, DI-water was used at 0.1 MPa. Secondly, 1.0 g/L BSA solution (pH = 7) was filtrated through cross-flow cell for 80 min at 0.1 MPa and the permeate flux (*J_p_*) was calculated. Thirdly, the cleaning of the fouled membrane was completed with DI-water for 20 min at a stirring speed of 600 rpm, and then the PWF (*J_R_*) of the membrane washed with DI-water was calculated again. The above-mentioned filtration cycle was repeated thrice for each membrane [40,41].

Equation (6) was used to compute the flux recovery ratio (FRR).

(6) $$FRR(\%)=(\frac{J_{R}}{J_{W}})\times 100\%$$

Three resistance parameters were characterized to make a thorough study on the membrane fouling behavior. The total fouling ratio (*R_t_*), irreversible fouling ratio (*R_ir_*) and reversible fouling ratio (*R_r_*) were computed by utilizing the following equations:

(7) $$R_{t}=(1-\frac{J_{P}}{J_{W_{}}})\times 100\%$$

(8) $$R_{r}(\%)=(\frac{J_{R}-J_{P}}{J_{W}})\times 100\%$$

(9) $$R_{ir}(\%)=(\frac{J_{W}-J_{R}}{J_{W}})\times 100\%$$

where *R_t_* is the total decrease in flux degree because of the total membrane fouling. While *R_r_* and *R_ir_* are the sum of *R_t_*, *R_ir_* is the fouling caused due to the deposition of foulant (protein) on the membrane surface and membrane pores, and *R_r_* is the fouling caused by concentration polarization. The permeate flux and rejection for BSA were performed at a subsequent time to assess the long-term stability of the fabricated membranes.

## 3. Results and Discussions

### 3.1. The Pore Size Distribution

**Figure 2:**
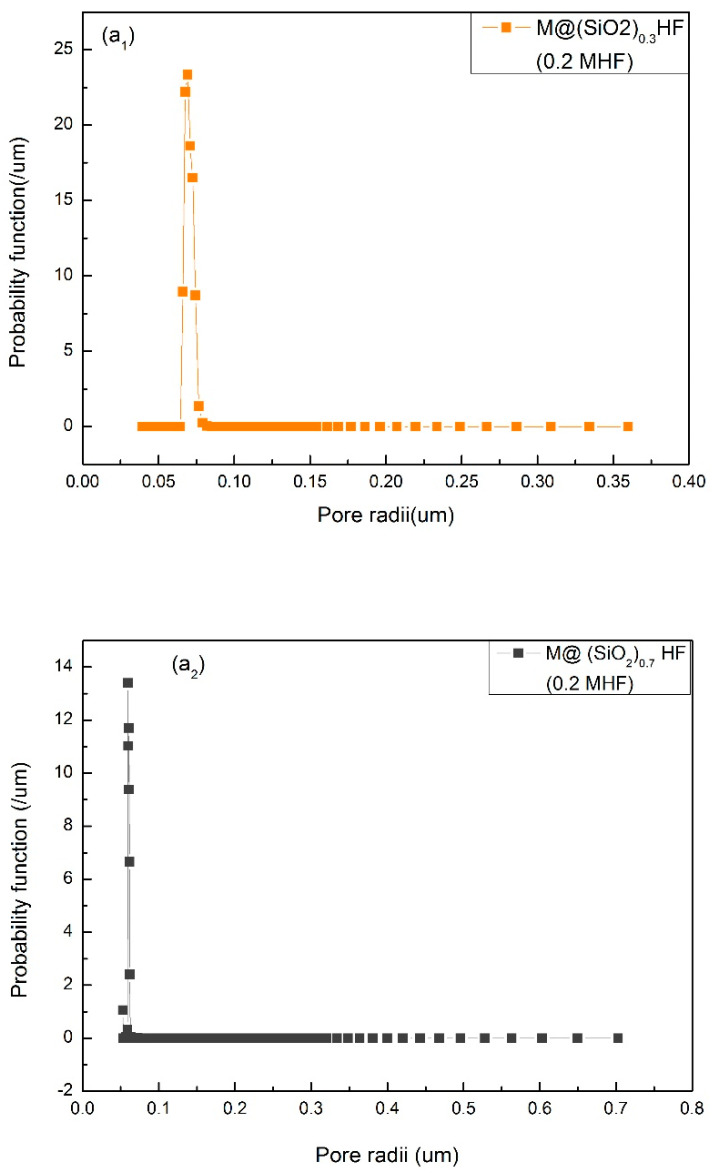

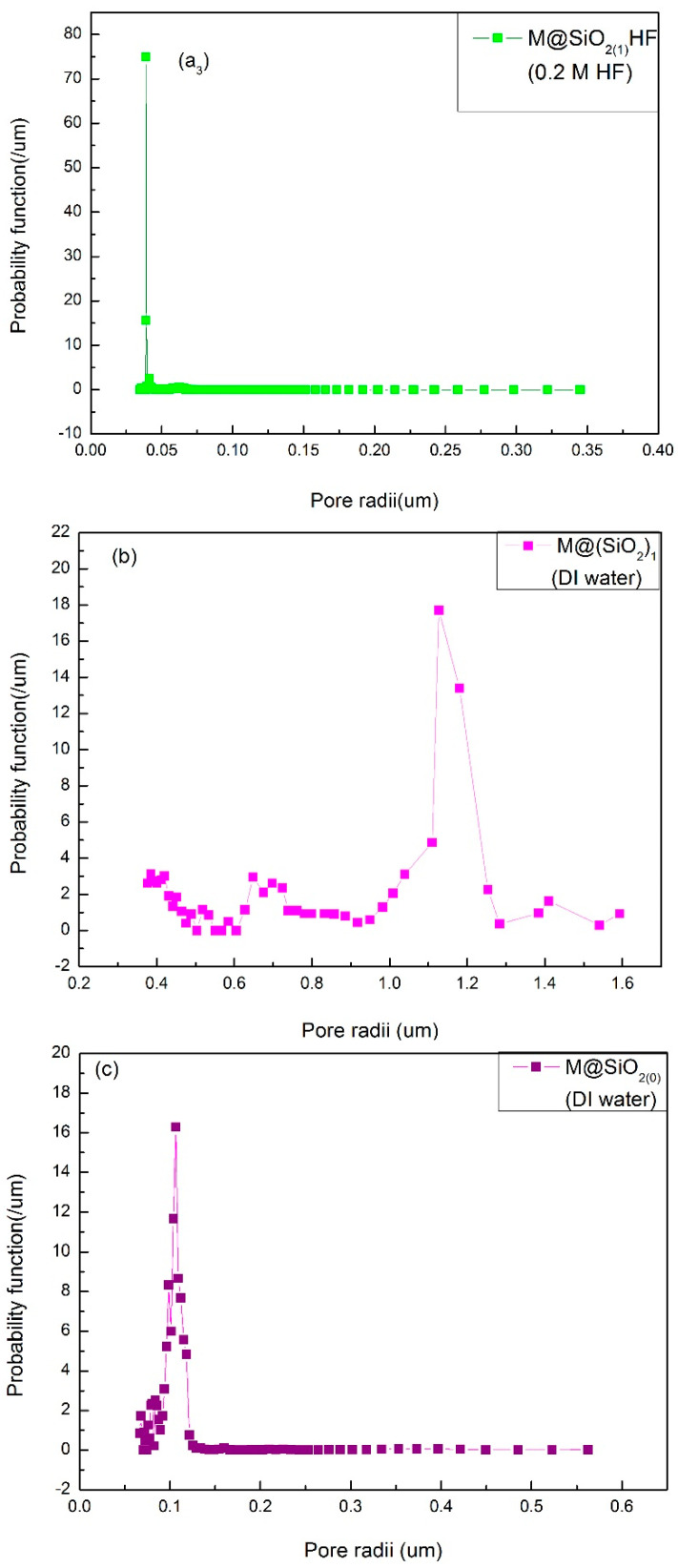

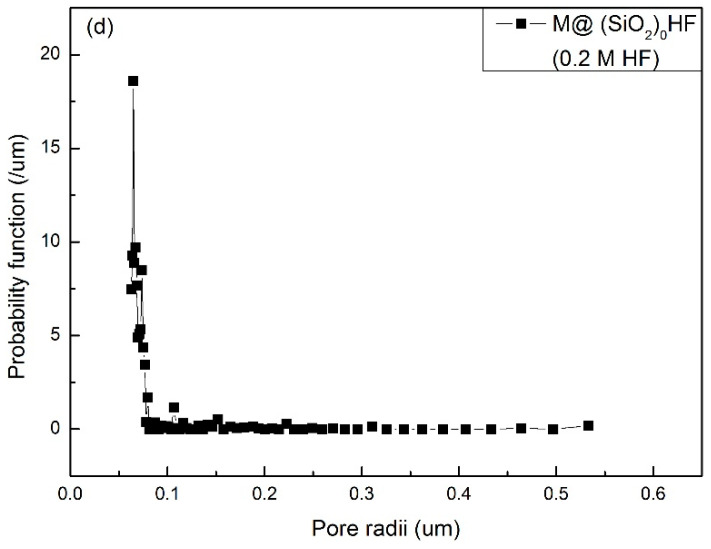
(**a_1_**–**a_3_**) are pore size distribution of M@SiO_2(0.3)_HF /M@SiO_2(0.7)_HF / M@SiO_2(1)_HF membranes with 0.2 M HF solution as a coagulant; (**b**) pore size distribution of M@SiO_2(1)_ membranes with DI-water as a coagulant; (**c**,**d**) pore size distribution of M@SiO_2(0)_HF/ M@SiO_2(0)_ membranes with 0.2 M HF/ DI-water as coagulation baths.

The membranes which possess a large number of pores with narrow mean pore radii and a uniform pore size distribution can exhibit superior filtration efficiency [4,5]. The mean pore radii and pore size distributions of the prepared membranes with different coagulation baths (0.2 M HF solution/DI-water) and different concentrations of SiO_2_NPs (0, 0.3, 0.7 and 1 wt%) are given in Figure 2. A sharp decrease in the mean pore radii (0.06 μm/0.05 μm/0.03 μm) of the etched membranes (M@SiO_2 (0.3)_HF/M@SiO_2 (0.7)_HF/M@SiO_2 (1)_HF) with an increase in SiO_2_ concentration can be seen in Figure 2a_1_, a_2_ and a_3_. This significant reduction in pore size is credited to the generation of pores/voids during etching mechanism. This indicates that an increase in SiO_2_NP concentration in the membrane matrix increased the number of pores, which resulted in high porosity and reduced mean pore size. 

On the other hand, the un-etched membrane M@SiO_2 (1)_ prepared with DI-water (coagulation bath) possesses bigger mean pore radii (1.16 μm) and its pore radius distribution range (1.16 ± 0.060 μm) is also broader (Figure 2b) than the etched membrane (0.030 ± 0.005 μm) with the same SiO_2_ concentration. Moreover, the pore size distribution of the former membrane is also more uniform and it had the smallest mean pore radii which resulted in higher flux. Contrastingly, the bigger mean pore radii (1.16 μm) in the case of the latter membrane resulted in less permeation due to clogging of pores by protein. When NPs were etched in the membrane matrix, pore connectivity and asymmetry were significantly improved. As a result, a well-connected microporous membrane with high porosity was obtained and its average pore size was reduced to around 0.03 μm. The mean pore size and porosity of the fabricated membranes are given in Table 1.

### 3.2. Surface Hydrophilicity

**Figure 3:**
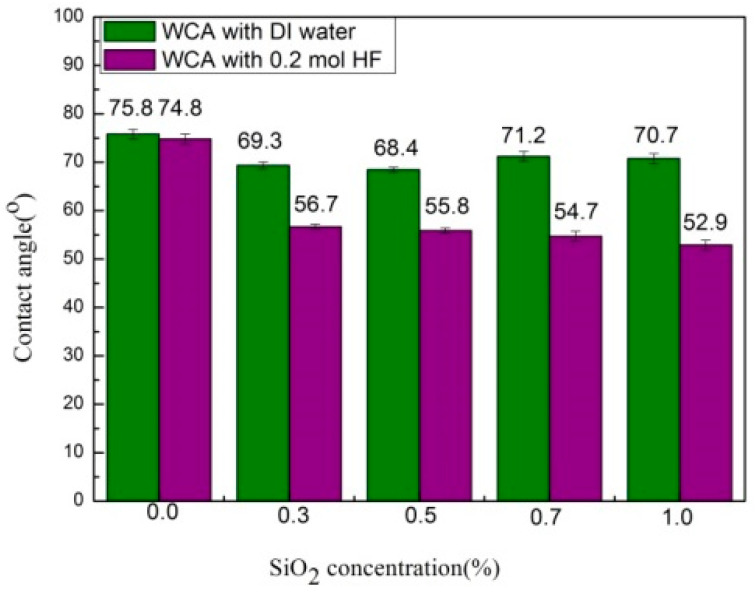
Water contact angles of the prepared membranes with a DI-water/0.2 M HF solution as a coagulation bath.

The surface hydrophilicity is always considered a crucial parameter for the performance of membranes, as it plays a vital role in enhancing the permeation and antifouling property. The membrane with superior hydrophilicity has the ability to adsorb more water molecules and resist the attachment of pollutants (e.g., macromolecules, ions, etc.) to its surface [35]. Therefore, the wettability of the prepared membranes is tested by measuring their water contact angles (WCA) (Figure 3). A decrease in water contact angle with an increase in SiO_2_NPs (0.3 to 1 wt %) is observed in the case of the etched membranes. Among all the prepared etched membranes, M@SiO_2 (1)_ HF exhibited the lowest WCA value of 52.9° due its excellent hydrophilicity and higher porosity (89%). As etching of SiO_2_NPs results in the formation of pores/voids, a larger number of voids were formed due to higher concentration of SiO_2_NPs in the membrane matrix which, in turn, increased the porosity with extra pore channels for water molecules to penetrate the membrane [42]. On the other hand, un-etched membranes M@SiO_2(0.7)_HF/M@SiO_2(1)_HF showed the highest WCA values (71.2° and 70.7°, respectively) due to the clogging of the valleys with SiO_2_ NPs, which were removed by HF in the case of the etched membranes. Thus, etching of SiO_2_NPs in the membrane matrix can significantly improve the membrane surface properties, which is vital for their application.

### 3.3. Morphological Investigation

**Figure 4:**
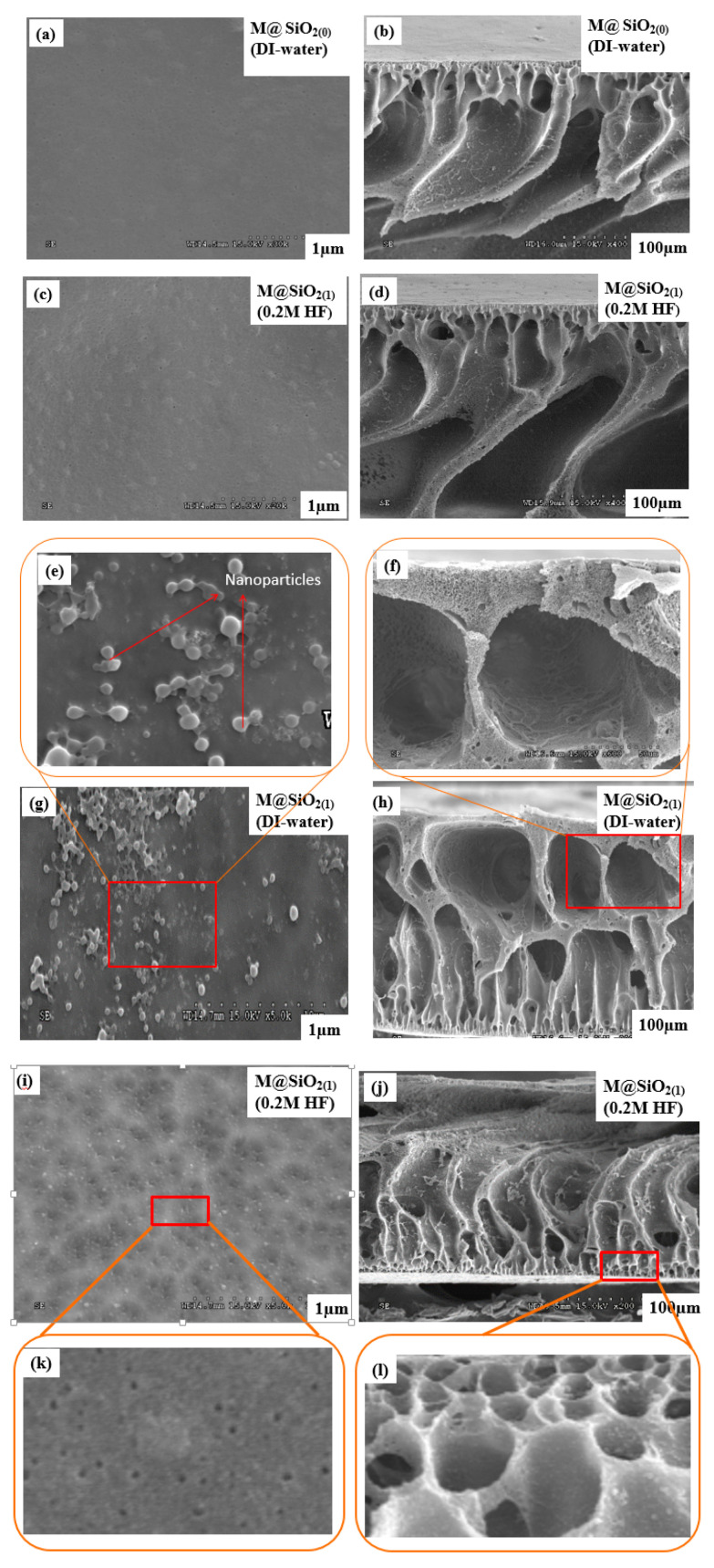
(**a**,**b**) are the surface and cross-sectional images of the membrane M@SiO_2(0)_ with DI-water as a coagulant; (**c**,**d**) are the surface and cross-sectional images of the membrane M@SiO_2(0)_HF with a 0.2 M HF solution as a coagulant; (**e**) is NPs on the surface of M@SiO_2(1)_ and (**f**) macro-voids near the surface of M@SiO_2(1)_; (**g**,**h**) are the surface and cross-sectional images of the membrane M@SiO_2(1)_ with DI-water as a coagulant; (**i**,**j**) are the surface and cross-sectional images of the membrane M@SiO_2(1)_HF with a 0.2 M HF solution as a coagulant; (**k**) is pores on surface of M@SiO_2(1)_HF and (**l**) is pores near the surface of M@SiO_2(1)_HF.

Surface and cross-sectional photographs of all the fabricated membranes are given in Figure 4. The results for the M@SiO_2(0)_ and M@SiO_2(0)_ HF membranes illustrate that both of the membranes possess a smooth surface without any visual defect. This indicates that the coagulant has no effect on the appearance of membrane surface in terms of pore size and porosity in the absence of SiO_2_ NPs in the membrane matrix. Similarly, cross-sectional observations reveal that both of the membranes also possess almost the same morphological structure. They have a typical asymmetric structure with a dense top surface and un-straight finger-like projections in the sub-layer. Similarity in the cross-sectional as well as surface observations of both membranes indicates that the hydrofluoric acid as a coagulant has no effect on the morphology of the membrane when the concentration of SiO_2_NPs is 0 wt%, and the structure remains well preserved. In contrast, after the incorporation of SiO_2_NPs (1 wt%) in the casting solution, the membrane was found to have a porous structure with the straight-through finger-like projections and macro-voids. The reason is mainly because of the accelerated solvent (NMP) and non-solvent (DI-water) exchange rate during the phase inversion process due to embedded hydrophilic SiO_2_ NPs in casting solution, which resulted in macro-voids and finger like pores [43]. On the other hand, the etching of the SiO_2_ NPs (1 wt%) in the membrane matrix using a 0.2 M HF solution as a coagulant significantly affected the morphology of the M@SiO_2(1)_ HF membrane. The M@SiO_2(1)_ HF membrane exhibited un-straight finger-like projections with a highly porous top surface due to the formation of voids/pores. These voids/pores are the result of chemical reaction between SiO_2_ NPs in the membrane matrix and the 0.2 M solution of HF in the coagulation bath which promoted the formations of narrower and large pore channels by accelerating the solvent and non-solvent exchange rate [44] and increased the thermodynamic instability of the cast film. As a consequence, the instantaneous de-mixing occurred in the coagulation bath, which resulted in un-straight-through finger-like projections, which provided the additional pathways for water transportation and increased the filtration efficiency [45]. Therefore, the etching mechanism does not destroy the structure of the membrane and its morphology is well preserved.

### 3.4. Performance of Blend UF Membranes

#### 3.4.1. Membrane Permeation Flux

**Figure 5:**
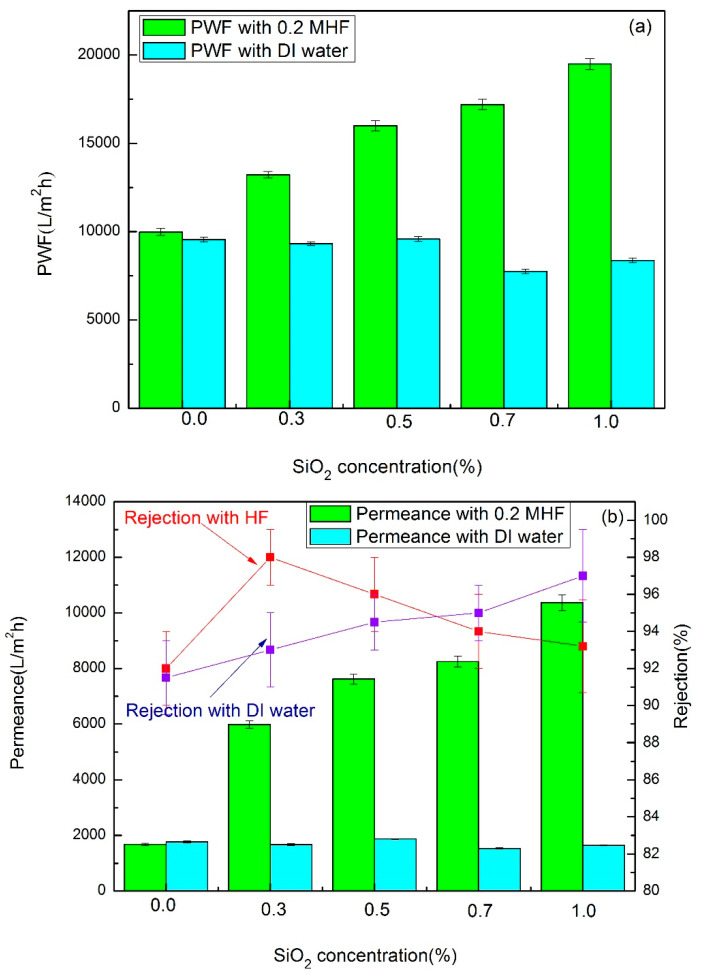
(**a**) PWF flux, (**b**) permeation flux and rejection of the etched and un-etched membranes prepared with 0.2 M HF/DI-water as coagulation baths.

The permeation through a membrane is very important for its application. Thus, the filtration experiments were performed to evaluate the permeation properties of the modified membranes. Figure 5 shows the pure water flux, permeation flux and BSA rejection rates of the prepared membranes with different coagulation baths (DI-water/ 0.2 M HF solution). 

As indicated in Figure 5a, by increasing SiO_2_ concentration from 0.3 to 1 wt% in the membrane matrix, the PWF through the un-etched membranes decreased. In contrast, an increase in PWF through the etched membranes is observed with increasing SiO_2_ concentration from 0.3 to 1 wt%.

This revealed that the etching of different SiO_2_ NP concentrations in the membrane matrix enhanced the porosity and provided the membrane with good hydrophilicity. Consequently, a higher pure water flux(PWF)of the etched membranes was found, and it was comparatively 2.3 times that of the un-etched membranes. Similarly, a rapid rise in permeate flux (PF) was noticed with rising values of SiO_2_ NP concentration (Figure 5b). For instance, a comparative rise in PF of etched membrane was 3.6 times higher with 0.3 wt% of SiO_2_ NP concentration, and it was 6.5 times higher when we further increased SiO_2_ NPs from 0.3 to 1 wt%. The probable cause is that the porous structure and uniform pore size distribution of the etched membranes provide additional pathways for water transportation and also decreases the tortuosity, which is in favor of PF. However, both PF and PWF have shown totally opposite trends the in case of un-etched membranes. The decline in both of these characteristics with a rise in SiO_2_ NP concentration is attributed to the agglomeration of SiO_2_ NPs, which may block the pathways [35]. It will result in the resistance for water transport. The flux and rejection of all the prepared membranes are presented in Table 2. 

To further investigate the increase in membrane permeability after the etching of SiO_2_ NPs in the membrane matrix, SEM images of the membranes were taken under consideration (Figure 4). The etching of SiO_2_ NPs in the membrane matrix resulted in more porous and asymmetric membrane structures. The asymmetric membranes are fouling-resistant, which is consistent with the increased PWF and higher rejection. A performance comparison of the current study with the literature is given in Table 3. 

#### 3.4.2. BSA Rejection Performance

Protein is the main problematic pollutant among other organic foulants of UF membranes [53]. As the BSA rejection rate reflects the size exclusion ability of the membrane surface, a relatively higher value of BSA rejection in the present study implies that the pore size distribution and geometry of the skin layer of the membrane has significantly changed. A comparison of BSA rejection for the etched and un-etched membranes is presented in Figure 5b.

The results show that the un-etched membranes exhibit a slightly higher rejection rate with an increase in SiO_2_ NPs, and the highest rejection ratio of 97% is achieved for M@SiO_2(1)_. This is associated with the less porous structure and relatively un-uniform pore size distribution, which prevents the penetration of BSA molecules through the membrane by reducing the number of paths [54].

Contrastingly, a slight decrease in BSA rejection of the etched membranes with an increase in SiO_2_ NPs is observed, and the lowest rejection ratio of 93.2% is achieved by M@SiO_2(1)_HF. This is attributed to the more and asymmetric pore channels formed by rapid phase inversion.

### 3.5. Antifouling Performance

The fouling of the membrane surface by a foulant can cause a considerable decline in the permeation flux. A reduction in hydrophobic attraction between the membrane surface and the foulant through hydrophilic surface modification can overcome the fouling problem [55,56]. 

**Figure 6:**
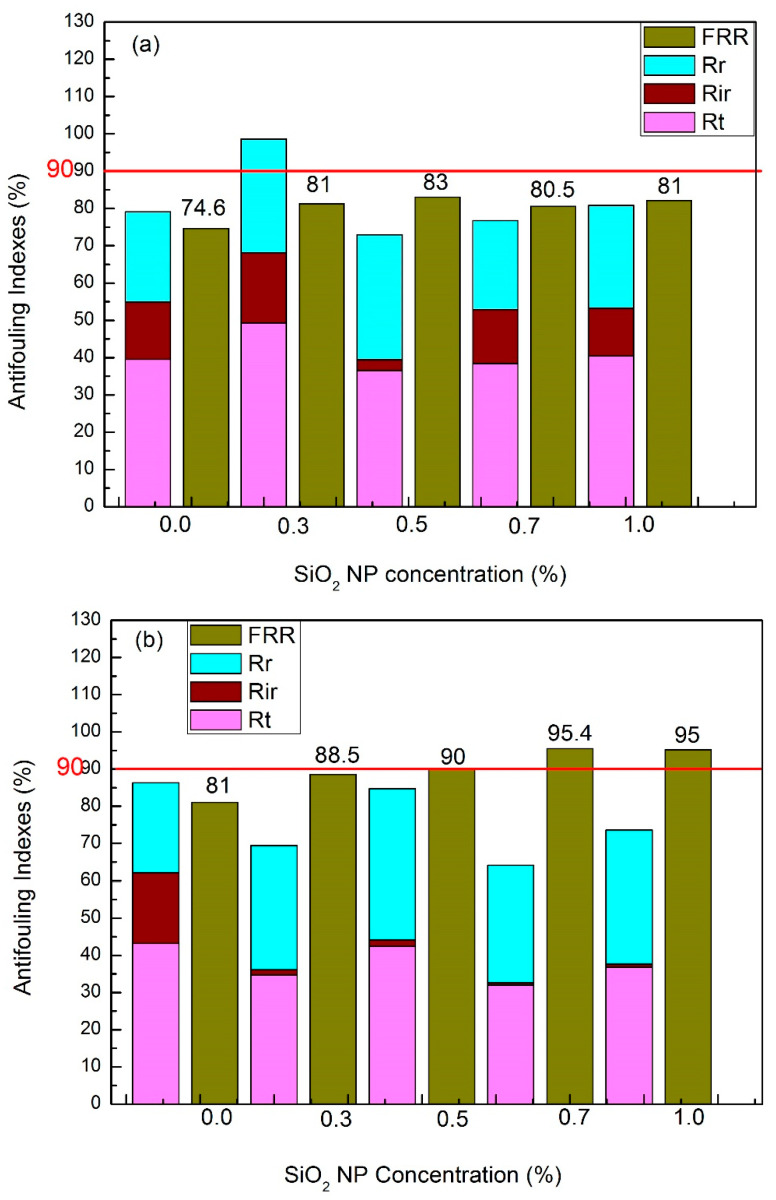

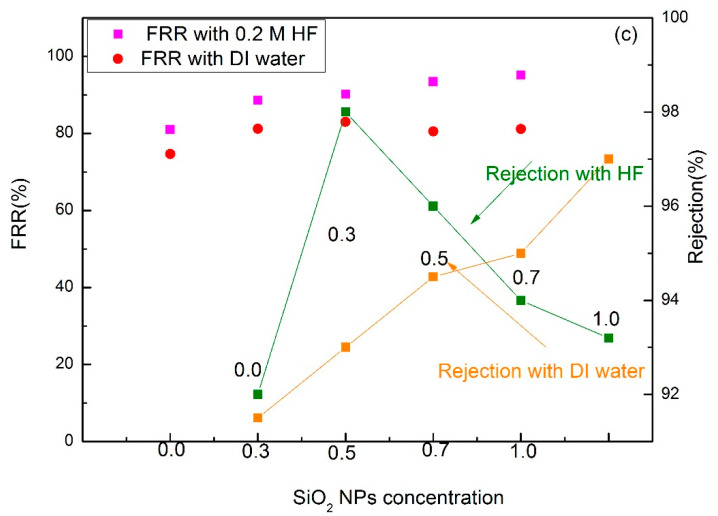
(**a**) Antifouling indexes of the un-etched membranes prepared with DI-water as a coagulant; (**b**) antifouling indexes of the etched membranes prepared with 0.2 M HF solution as a coagulation bath; (**c**) rejection and FRR of the etched and un-etched membranes.

The antifouling capacity of all the synthesized membranes is tested and the corresponding results are given in Figure 6. The evaluation of the membrane antifouling property flux recovery ratio (FRR) is a crucial parameter, and the membranes with a higher FRR value possess excellent antifouling performance. However, a significant improvement in the antifouling capacity of the etched membranes was observed (Figure 6b). As seen in Figure 6b, the FRR of the etched membranes sharply increased with an increase in SiO_2_NP concentration in the membrane matrix. The maximum FRR is 95% (81% for without etching), which was witnessed for the membrane M@SiO_2 (1)_ HF. This indicates that the higher filtration efficiency can be achieved after etching SiO_2_NP in the membrane matrix by HF solution as it generates the pores/voids in the membrane structure, which increase with an increase in SiO_2_NP concentration. Therefore, the membrane structure becomes more porous, which increases the hydrophilicity of the prepared membrane. As a consequence, more water molecules are attracted to its surface to create a compact water barrier which prevents the interactions between foulants and the membrane surface [46]. To further investigate the antifouling performance of the prepared membranes, fouling resistance was tested, which includes the irreversible resistance (*R_ir_*_)_ and reversible fouling (*R_r_*).The results of fouling parameters (*R_ir_*) and (*R_r_*) are shown in Figure 6a,b.

A remarkable decrease in the *R_ir_* (0.5%/0.8%) was observed for the etched membranes M@SiO_2(0.7)_HF and M@SiO_2(1)_HF, and this is attributed to the superior features of the membrane surface properties (higher hydrophilicity and higher porosity), which can weaken the interactions between the membrane surface and foulants by reducing the contact area [47]. On the other hand, a sharp decline in permeation flux for the un-etched membranes with an increase in SiO_2_ NP concentration was observed due to membrane fouling (Figure 5b). The un-etched membranes exhibited higher irreversible fouling because of the less porous sub-layer structure and less hydrophilicity. Adsorption of foulants on the membrane surface and in the valleys of the membrane is caused due to interactions between the membrane surface and foulants [43], and this ultimately results in the clogging of the valleys that cannot be washed simply by rinsing.

## 4. Conclusion

In the current study, a highly porous UF membrane with a narrow (0.03 μm) and more uniform pore size distribution (0.030 ± 0.005 μm) has been successfully synthesized via the combination of the etching mechanism and the L-S phase inversion method for the first time. Different concentrations of SiO_2_ NPs in the membrane matrix were etched with a 0.2 M HF solution in a coagulation bath. The etching mechanism engendered voids/pores which provided the membrane with a hydrophilic and highly porous surface. As a consequence, the prepared etched MF membrane exhibited a rejection of 93.5%, with high PWF (19494 L/m^2^·h) and enhanced permeance (10,368 L/m^2^·h). Moreover, the membrane achieved an excellent antifouling property (i.e., FRR of 95% for a 1000 mg/L BSA aqueous solution). It can be concluded that an etched UF membrane with asymmetric and a reduced mean pore radii (0.03 μm) can be prepared via the etching mechanism combined with the L-S phase inversion method, which significantly improves the pore size distribution and porosity. Hence, the proposed method of etching SiO_2_ nano-particles in membrane matrix may be considered as a feasible approach to improve the structure and separation efficiency of the PVDF/PMMA/CA blend membrane.

## Figures and Tables

**Table 1:** Porosities and mean pore size of the PVDF/PMMA/CA membranes.prepared with different coagulation baths (DI-water and HF solution).

Sample (16%)	SiO2 NPs (wt%)	Coagulation Bath	Porosity (%)	Mean Pore Size (µm)
M@SiO_2(0)_	0	DI-water	77	0.10 ± 0.070
M@SiO_2(0)_ HF	0	0.2 M HF(aq)	75	0.09 ± 0.010
M@SiO_2(1)_	1	DI-water	78	1.16 ± 0.060
M@SiO_2(1)_ HF	1	0. 2M HF(aq)	89	0.03 ± 0.005

**Table 2:** PWF, ptmeation flux, and rejection of all modified membranes.

PVDF/PMMA/CA (%) 8.2/2.4/2.4	Solvent	SiO_2_NPs (wt%)	PVP	Coagulation Bath	PWF (L·m^−2^·h^−1^)	Permeance (L·m^−2^·h^−1^)	Rejection _BSA_ (%)	Contact Angle (°)
M@SiO_2(0)_	NMP	0	3	DI-water	9555.0 ± 195	1766 ± 35	91.5 ± 1.5	75.81 ± 1.0
M@SiO_2(0)_ HF	NMP	0	3	0.2 M HF(aq)	9555.0 ± 195	1681 ± 40	92.0 ± 1.0	74.82 ± 1.0
M@SiO_2(0.3)_	NMP	0.3	3	DI-water	9323.0 ± 190	1670 ± 30	93.0 ± 0.6	69.36 ± 0.7
M@SiO_2(0.3)_HF	NMP	0.3	3	0.2 M HF(aq)	13,216.0 ± 109	5985 ± 134	98.0 ± 1.0	56.70 ± 0.5
M@SiO_2(0.5)_	NMP	0.5	3	DI-water	9586.0 ± 278	1872 ± 15	94.5 ± 1.5	68.46 ± 0.5
M@SiO_2(0.5)_HF	NMP	0.5	3	0.2 M HF(aq)	15,995.0 ± 145	76206 ± 176	96.0 ± 1.0	55.88 ± 1.0
M@SiO_2(0.7)_	NMP	0.7	3	DI-water	7748.0 ± 298	1533 ± 23	95.0 ± 0.7	71.22 ± 1.0
M@SiO_2(0.7)_ HF	NMP	0.7	3	0.2 M HF(aq)	17,197.0 ± 120	8246 ± 200	94.0 ± 0.5	74.74 ± 1.0
M@SiO_2(1)_	NMP	1.0	3	DI-water	8374.0 ±134	1640 ± 17	97.0 ± 0.3	70.78 ± 1.0
M@SiO_2(1)_HF	NMP	1.0	3	0.2 M HF(aq)	19,494.0 ± 300	10,368 ± 89	93.5 ± 1.0	52.91 ± 1.0

Note: PVDF, PMMA, CA, PVP and SiO_2_ stand for polyvinylidene fluoride, polymethyl acrylate, cellulose acetate, polyvinylpyrrolidone and silicon oxide, respectively.

**Table 3:** Performance comparisons of the prepared membrane with the literature.

Membrane	Water Flux (L/(m^2^.h))	Rejection (%)	Contact Angle (°)	FRR (%)	Reference
PVDF/GO–SiO_2(0.5 wt%)_(MF)	850	92.0	68.0	62	[46]
CA/MOF@GO_0.12_	183.51	93.3	49.5	88	[1]
PVDF/GO _(1 wt%)_ (UF)	505	87.0	68.0	74	[47]
PVDF/GO/MWCNTs _(1 wt%)_ (UF)	406	-	52.0	98	[48]
PVDF/GO _(2 wt%)_ (UF)	25	-	64.0	80	[49]
PVDF/rGO/TiO _2 (0.05 wt%)_ (UF)	221	99.0	69.0	95	[50]
PVDF/GO _(3 wt. %)_ (MF)	505	93.0	61.0	-	[51]
CA/PVP_(3 wt. %)_	978	77.9	47.3	85.2	[43]
CA/GA/ Na_2_CO_3 (aq). (2 wt.%)_	2836	90.8	-	-	[52]
PVDF/PMMA/CA/SiO_2(1wt %)_	10,368	93.5	52.9	99	Present Study

## Nomenclature

*A*	effective area of the membrane (m^2^)	*t*	filtration time (h)
*Cf*	concentrations of the feed solution (g/L)	*FR*R	rejection ratio (%)
*Cp*	concentrations of the permeate solution (g/L)	*R*	rejection ratio (%)
*J*	permeate flux (L/m^2^·h)	*R_t_*	total fouling ratio (%)
*J_p_*	flux of BSA (L/m^2.^h)	*R_ir_*	irreversible fouling ratio (%)
*J_R_*	flux of fouled membrane washed with DI-water (L/m^2^h)	*R_r_*	reversible fouling ratio (%)
